# Influence of Thermal Treatment on the Antimicrobial Activity of Silver-Doped Biological Apatite

**DOI:** 10.1186/s11671-015-1211-x

**Published:** 2015-12-29

**Authors:** Cristina Liana Popa, Carmen Steluta Ciobanu, Georgeta Voicu, Eugenia Vasile, Mariana Carmen Chifiriuc, Simona Liliana Iconaru, Daniela Predoi

**Affiliations:** National Institute of Materials Physics, 105 bis Atomistilor Street, Măgurele, Romania; Department of Science and Engineering of Oxide Materials and Nanomaterials, University Politehnica of Bucharest, Faculty of Applied Chemistry and Materials Science, 1-7 Polizu Street, Bucharest, Romania; Microbiology Department, Faculty of Biology, University of Bucharest, Aleea Portocalelor 1-3, 60101 Bucharest, Romania; Research Institute of the University of Bucharest—ICUB, Life, Environmental and Earth Sciences, Spl. Independentei 91-95, Bucharest, Romania

**Keywords:** Silver, Hydroxyapatite, Thermal treatment, Antimicrobial effect

## Abstract

In this paper, we report the structural and morphological properties of silver-doped hydroxyapatite (AgHAp) with a silver concentration *x*_Ag_ = 0.5 before and after being thermal treated at 600 and 1000 °C. The results obtained by X-Ray diffraction (XRD), Fourier transform infrared spectroscopy (FTIR), and Raman spectroscopy suggest that the structure of the samples changes gradually, from hydroxyapatite (AgHAp_40) to a predominant β-TCP structure (AgHAp_1000), achieved when the thermal treatment temperature is 1000 °C. In the AgHAp_600 sample, the presence of two phases, HAp and β-TCP, was highlighted. Also, scanning electron microscopy studies suggest that the shape and dimension of the nanoparticles begin to change when the temperature increases. The antimicrobial activity of the obtained compounds was evaluated against *Klebsiella pneumoniae*, *Staphylococcus aureus*, and *Candida albicans* strains.

## Background

In the last decade, the materials based on calcium phosphate, such as hydroxyapatite and tricalcium phosphate, have received special attention from the viewpoint of theoretical and experimental research. These materials have attracted the interest of researchers from the biomedical field due to the fact that they possess excellent biological properties (bioactivity, biocompatibility, osseoconductivity, etc.) which recommend them for different applications in stomatology, orthopaedics, as well as in other medical fields [1–4].

Hydroxyapatite (HAp, Ca_5_(PO_4_)_3_(OH)), which belongs to the calcium phosphate family, is one of the most studied biomaterials. It is the main inorganic component of bone tissue [2–4]. Its unique structure allows it to be doped with an important number of metallic ions, such as Ag^+^, Eu^3+^, Mg^2+^, F^−^, and Cu^2+^ [1–4]. Usually, these kinds of substitutions help to improve its biological properties. On the other hand, one of the most important properties of HAp, when referring to implants fabrication usually requiring high temperatures, is its thermal stability [5–8].

Previous studies [9–12] have reported that doping hydroxyapatite with silver ions led to their incorporation in the HAp structure by substituting Ca^2+^ ions. As a result of this process, the standard molar ratio Ca/P of the samples changed, thus the stoichiometry of the samples was lost. In this context, the thermal treatment at 600 and 1000 °C leads to the formations of secondary phases: β-tricalcium phosphate (*T* > 700 °C, β-TCP) or α-tricalcium phosphate (*T* > 1200 °C, α-TCP) [9–15]. These structural changes due to temperature strongly influence the physicochemical and biological properties of hydroxyapatite. Both compounds (β-TCP and α-TCP) are known as being osseoinductive and biocompatible [13–15].

Nevertheless, the excellent biocompatibility with the bone tissue strongly recommends hydroxyapatite as a biomaterial used in implantology, as well as for other clinical applications [16, 17].

In the case of dental and bone implantology, HAp facilitates the proper development of bone implant chemical and mechanical interface, acting as a bridge between implant and the receptor bone, due to their similar structure [18–20]. An important role in the implant failure is held by microbial infections. Soon after implantation, the implant surface is covered with a host-derived, conditioning pellicle that would favor the microbial adherence and the further development of a biofilm. The microbial cells and/or their soluble products induce an inflammatory response due to the installation of peri-implantitis in the soft tissues, affecting the osseointegration and leading to implant failure [16].

Silver has been long time known for its intrinsic microbiostatic or microbicidal properties, due to the ability of silver ions to interfere with the microbial membrane, affecting its structural integrity, DNA replication, cellular division, and protein activity. The silver nanoparticles exhibit a more intensive antimicrobial effect, depending on their size, shape, and dose [21].

However, the use of silver for antimicrobial applications could be significantly improved by the use of an effective drug delivery system [22]. Therefore, combining the naturally occurring antimicrobial properties of silver with the high biocompatibility and potential of HAp to be used for drug delivery could result in a promising bioactive system for biomedical applications with good antibiofilm and bone bonding capacity [23].

Therefore, the purpose of this study was to investigate the influence of the thermal treatment on the physicochemical and antimicrobial properties of the AgHAp powders.

Silver-doped hydroxyapatite with calcium deficiency obtained by co-precipitation method at room temperature, dried at 40 °C (AgHAp_40), followed by thermal treatment at 600 °C (AgHAp_600) and 1000 °C (AgHAp_1000) were investigated by X-ray diffraction (XRD), scanning electron microscopy (SEM), Fourier transform infrared spectroscopy (FTIR), and Raman spectroscopy. The antimicrobial activity of AgHAp_40, AgHAp_600, and AgHAp_1000 samples was evaluated using *Klebsiella pneumonia* 11, *Staphylococcus aureus* ATCC 6538, and *Candida albicans* ATCC 10231 strains.

## Methods

### Sample Preparation

The silver-doped hydroxyapatite with calcium deficiency powders (AgHAp, *x*_Ag_ = 0.5) was obtained by a co-precipitation method reported in another paper [2]. The Ag concentration was calculated from Ag/(Ca + Ag)*x*100 where *x* is related to the apatite formula Ca_10 − *x*_ Ag_2*x*_(PO_4_)_6_(OH)_2_. The initial solution based on calcium was modified to contain 50 % silver nitrate and 50 % calcium nitrate. The solution was kept at 40 °C and the pH at 10 during crystallization. The precipitate was stirred at 40 °C for 60 min and left in the mother solution for 24 h. Afterwards, the precipitate was filtered and washed several times with deionized water. The resulting material (AgHAp, *x*_Ag_ = 0.5) was dried at 40 °C and have been referred to as AgHAp_40. The AgHAp_40 powders were thermally treated at 600 °C (AgHAp_600) and 1000 °C (AgHAp_1000) for 8 h. Finally, the samples were milled in order to obtain fine powders.

### Characterization Methods

#### X-Ray Diffraction

The X-ray diffraction investigations were obtained using a Bruker D8 Advance diffractometer, with nickel filtered Cu K (λ = 1.5418 Å) radiation and the diffraction patterns were recorded between 20° and 70° in the 2*θ* range with a step of 0.02° and 34 s measuring time per step.

#### Scanning Electron Microscopy

Studies regarding the structure and morphology of the samples were performed using a Quanta Inspect F50 microscope, with a field emission gun (FEG) equipped with an energy dispersive X-ray attachment. The energy dispersive X-ray (EDAX) analysis was used to identify the elemental composition of the materials.

#### FTIR Spectroscopy

Information about the functional groups present in the prepared powders were obtained using an attenuated total reflectance Fourier transform (ATR-FTIR) spectrophotometer SP 100 PerkinElmer. Each spectrum was acquired in transmittance mode on a Diamond/KRS-5 crystal cell with a resolution of 4 cm^−1^ and a wave number range between 4000 and 400 cm^−1^. Further signal processing included the transformation from transmittance to absorption. The spectra are presented in this paper in absorption mode.

#### Raman Spectroscopy

Raman studies were performed using a LabRAM HR Evolution from Horiba (Jobin Yvon) equipped with a nitrogen-cooled detector, under laser excitation wavelength 633 nm produced by a helium-neon laser. The spectra were recorded in a spectral range 200–4000 cm^−1^.

#### Antimicrobial Activity Assays

The antimicrobial activity of the obtained compounds was assessed against Gram-negative (*K. pneumoniae* 11) and Gram-positive (*S. aureus* ATCC 6538) bacterial and fungal (*C. albicans* ATCC 10231) strains.

Microbial suspensions of 1.5 × 10^8^ CFU mL^−1^ (0.5 McFarland density) obtained from 15 to 18 h of bacterial cultures developed on solid media were used. The tested powders were suspended in dimethyl sulfoxide (DMSO) to prepare a stock solution of 10 mg/mL^−1^ concentration. The antimicrobial activity was tested on Mueller-Hinton Agar (MHA). The qualitative screening was performed by an adapted disc diffusion method as previously reported [24]. The quantitative assay of the antimicrobial activity was performed by liquid medium microdilution method in 96 multi-well plates. Twofold serial dilutions of the compound solutions (ranging between 1 and 0.031 mg/mL) were performed in a 200 μL volume of broth, and each was well was seeded with 50 μL of microbial inoculum. Culture positive controls (wells containing culture medium seeded with the microbial inoculum) were used. The plates were incubated for 24 h at 37 °C, and the minimal inhibitory concentration (MIC) values were considered as the lowest concentration of the tested compound that inhibited the growth of the microbial overnight cultures, as compared to the positive control, revealed by a decreased value of absorbance at 620 nm (Apollo LB 911 ELISA reader) [25, 26].

## Results and Discussion

The present study investigates by using several techniques the influence of the temperature on the structure, morphology, and biological properties of silver-doped calcium-deficient hydroxyapatite (Ca_10 − *x*_Ag_*x*_(PO_4_)_6_(OH)_2_, AgHAp) with *x*_Ag_ = 0.5. For understanding the influence of the thermal treatment on the structure, morphology, and biological properties, the samples were analyzed using several techniques.

**Fig. 1:**
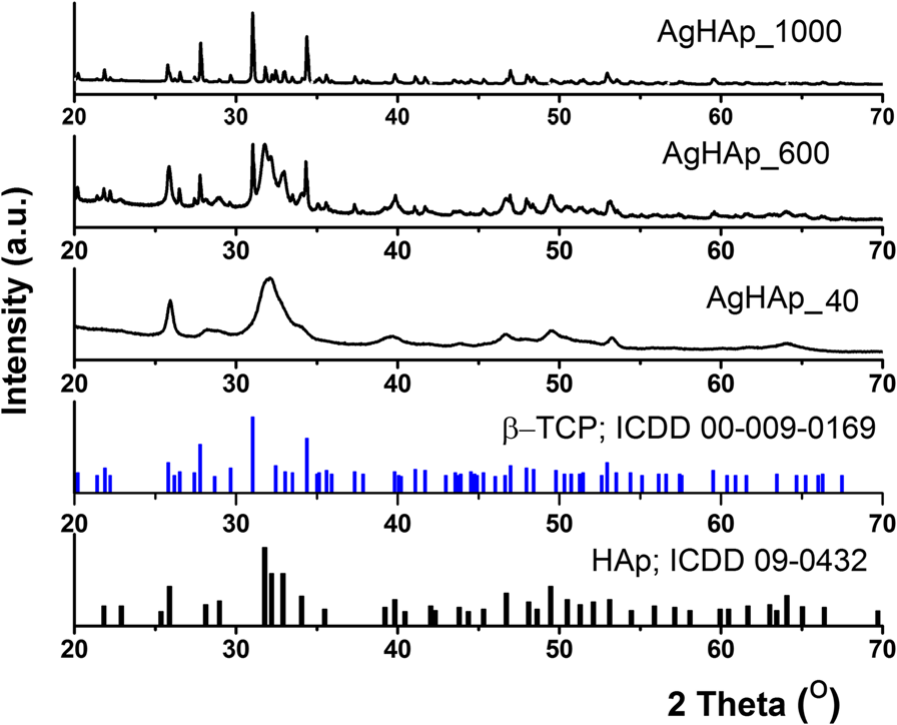
X-ray diffraction patterns for the different powders obtained at 40 °C (AgHAp_40) and after thermal treatment at 600 °C (AgHAp_600) and 1000 °C (AgHAp_1000). The standard ICDD PDF 09–0432 of hexagonal HAp and ICDD PDF 009–0136 rhombohedral of β-TCP are also presented for comparison

The X-ray diffraction (XRD) analysis of AgHAp dried at 40 °C and after the thermal treatment at 600 °C (AgHAp_600) and 1000 °C (AgHAp_1000) is presented in Fig. 1. At the bottom of the figure, as reference, the Powder Diffraction File (PDF) standard cards of pure hexagonal hydroxyapatite (ICDD 09–0432) and rhombohedral-tricalcium phosphate (ICDD 009–0136) are represented. The successful incorporation of silver ions in the HAp structure (sample AgHAp_40) was proved by the XRD phase analysis according to previous studies [21]. As seen from the figure, the XRD pattern of AgHAp_40 sample is typical for a hydroxyapatite with calcium deficiency. This result is in good agreement with the previous studies conducted by Berzina-Cimdina and Borodajenko [9].

It can be seen that by increasing the thermal treatment temperature, for both AgHAp_600 and AgHAp_1000 samples, the XRD analysis showed a biphasic material of pure hexagonal hydroxyapatite (HAp) and rhombohedral β-tricalcium phosphate (β-Ca_3_(PO_4_)_2_, β-TCP). To highlight the phase composition, Rietveld refinement studies were performed on the AgHAp samples at room temperature and after thermal treatment at 600 and 1000 °C. The MAUD software [27, 28] was used for the Rietveld refinements. The peaks of β-TCP appear more clear and well defined for the AgHAp_1000 sample. The β-TCP phase increased from 22 % (AgHAp_600) to 75 % for the sample thermally treated at 1000 °C (AgHAp_1000). It can be observed that the AgHAp samples thermally treated at 600 and 1000 °C consist of HAp and β-TCP phases. The results are consistent with the standard data PDF file number 09–0432 (HAp) and PDF file number 009–0136 (β-TCP). Increasing the thermal treatment temperature from 40 to 600 and 1000 °C was conducted to changes in the composition and crystallinity of the material.

**Fig. 2:**
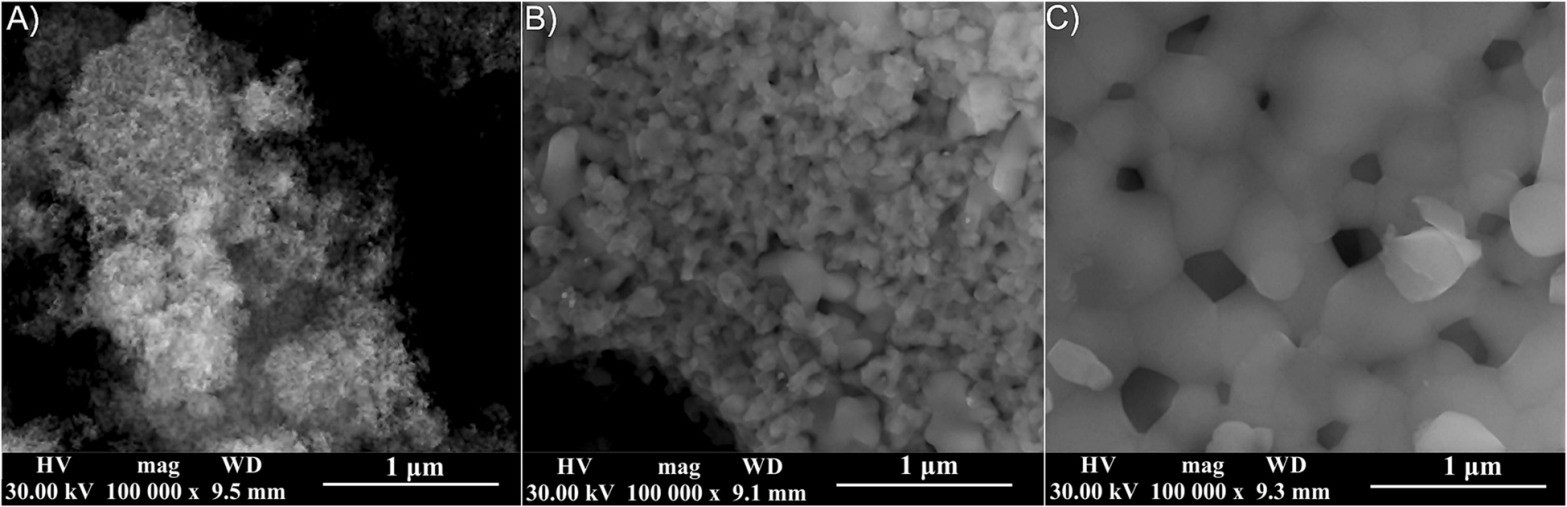
SEM images of AgHAp (*x*_Ag_ = 0.5) powders at 40 °C (**a**) and thermally treated at 600 °C (**b**) and 1000 °C (**c**)

The morphology of the studied powders was investigated by scanning electron microscopy. The results obtained by SEM are presented in Fig. 2. In these images, the influence of thermal treatment temperature on the morphology of the powders is highlighted. In the case of the AgHAp_40 sample (Fig. 2a), the nanoparticles exhibited an acicular morphology and tend to agglomerate.

The nanoparticle shape and size began to change when the thermal treatment temperature increased. In Fig. 2c (AgHAp_1000, *x*_Ag_ = 0.5), it can be observed on one hand the formation of grains and on the other hand the spherical shape of the nanoparticles. Moreover, the SEM micrographs confirm the increase of the nanoparticle dimensions with the increase of the thermal treatment temperature.

**Fig. 3:**
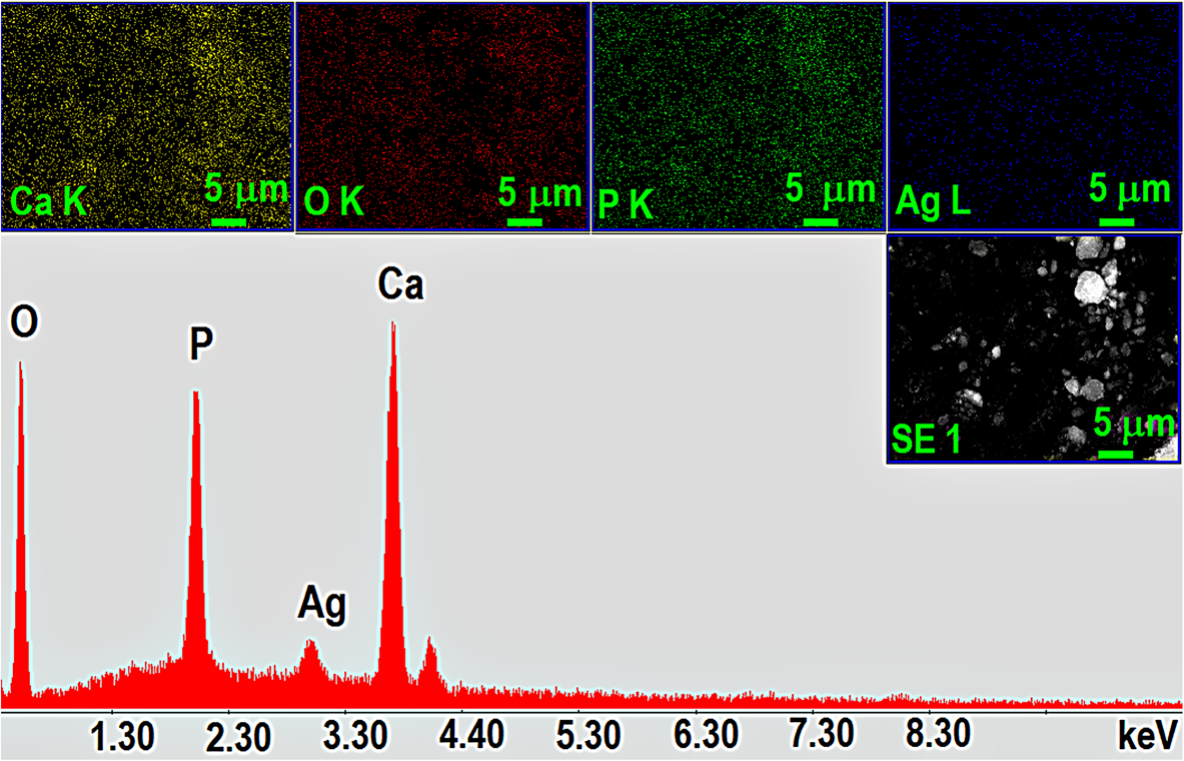
EDAX spectrum and elements mapping obtained for the AgHAp_40 (*x*_Ag_ = 0.5) powder

The EDAX spectrum (Fig. 3) obtained for the synthesized AgHAp powder (AgHAp_40) revealed the presence of the following chemical elements: Ca, P, Ag, O. All these elements make up the composition of AgHAp (*x*_Ag_ = 0.5) powder.

The homogenous and uniform distribution of the Ca, P, Ag, and O in the powders was highlighted by the elemental mapping (Fig. 3) obtained for the AgHAp_40 sample. The experimental concentrations (wt.%) of calcium and phosphorus in the prepared samples determined by inductively coupled plasma-atomic emission spectrometry (ICP-AES) are reported in Table 1. The experimental concentration (wt. %) of silver in the analyzed samples determined by ICP-AES was 4.693 ± 0.19. The atomic ratio Ca/P decreased from 1.66 for AgHAp_40 at 1.63 and 1.58 for AgHAp_600 and AgHAp_1000, respectively.

**Table 1:** Experimental concentrations (wt. %) of calcium and phosphorus in AgHAp samples

Samples	AgHAp_40	AgHAp_600	AgHAp_1000
Ca	38.02 ± 0.98	37.65 ± 0.88	33.98 ± 0.91
P	17.46 ± 0.38	18.35 ± 0.21	16.62 ± 0.12

**Fig. 4:**
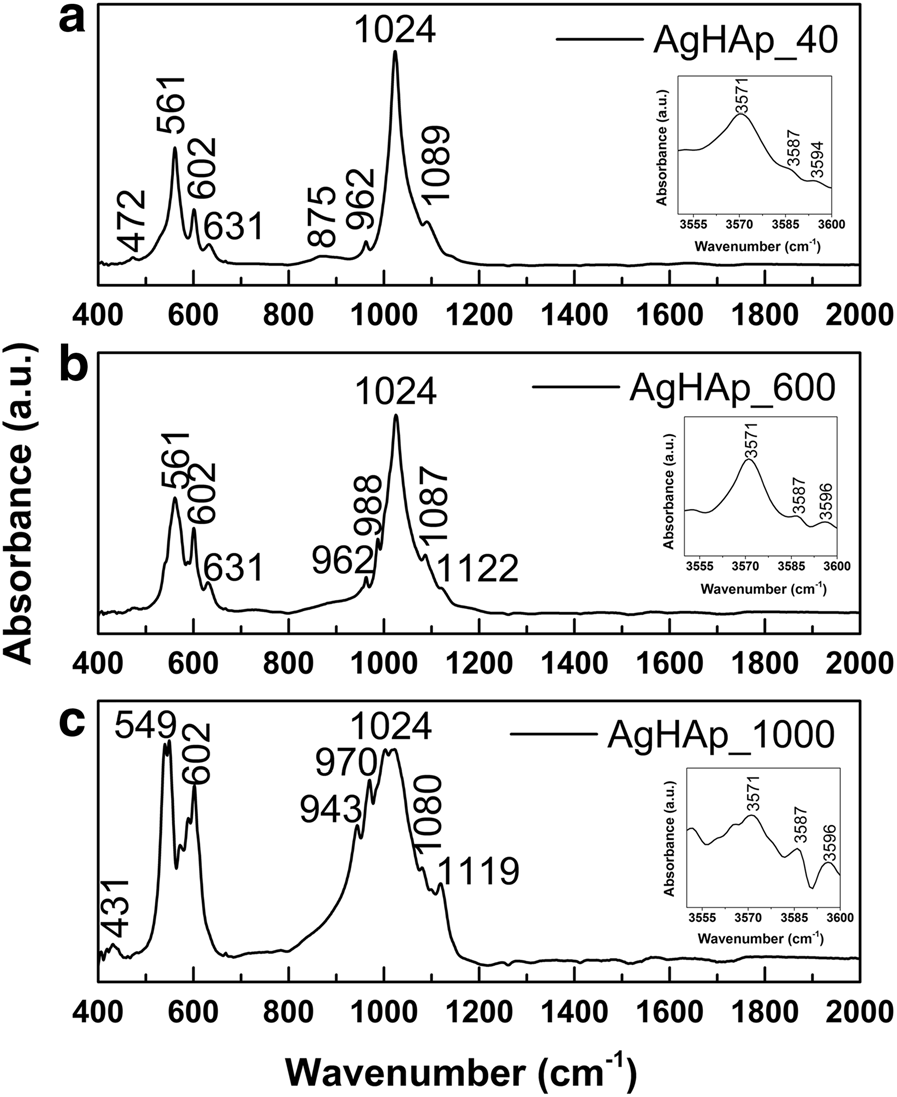
Infrared absorption spectra of the AgHAp (x_Ag_ = 0.5) before thermal treatment (**a**) and after the thermal treatment at 600 °C (**b**) and 1000 °C (**c**). The bands attributed to the water lattice (3550–3600 cm^−1^) are presented in the *top right corner* of each spectrum

In Fig. 4, the three infrared absorption spectra obtained for the silver-doped hydroxyapatite with *x*_Ag_ = 0.5 before the thermal treatment (Fig. 4a) and after the thermal treatment at 600 °C (Fig. 4b) and 1000 °C (Fig. 4c) are presented.

The spectrum in Fig. 4a exhibits the absorption bands characteristic to the structure of AgHAp_40 powder. The presence of peaks associated to phosphate, carbonate, and hydroxyl groups was evidenced. Therefore, the bands found at 472, 561, and 602 cm^−1^ are associated with the bending modes of the O-P-O bonds of the PO_4_^3−^ functional group [29–32]. Furthermore, the bands from 962 to 1024 cm^−1^ are characteristic to the stretching vibrations of the phosphate group [30–36]. The peak found at 1089 cm^−1^ is also characteristic to the vibration modes of the phosphate group [37]. The presence of the hydroxyl functional group in the structure of the AgHAp_40 sample is emphasized by the band found at 631 cm^−1^ which is associated to the librational mode [33, 38, 39]. The band from around 875 cm^−1^ is characteristic to the vibrations of the CO_3_^2−^ ions caused either by the absorption of carbon dioxide from the atmosphere during the synthesis or by impurities found in the sample [37]. The adsorbed water is evidenced by the presence of small, very wide bands in the 1600–1700 cm^−1^ spectral region [1].

After the thermal treatment of AgHAp_40 powder at 600 °C (Fig. 4b), the structure of the sample has begun to change. The main peaks from 561 to 602 cm^−1^ associated to the bending vibrations of the phosphate group [30–33] and the one from 1024 cm^−1^ associated to the stretching vibrations of the phosphate group [36] are still present. The peak found at 631 cm^−1^ which evidences the librational mode of the hydroxyl group [33, 38, 39] is also observed. However, an increase of the intensity of the 602 cm^−1^ peak is observed as well as a widening of the band found at 875 cm^−1^. This widening makes the band to be undistinguishable. The band which characterizes the stretching vibrations of the phosphate group previously found at 1089 cm^−1^ appears to have shifted slightly, to 1087 cm^−1^. On the other hand, two additional bands appear in the spectrum. The first one, found at 988 cm^−1^, is associated to the HPO_4_^2−^ [40] and the second one, from 1122 cm^−1^, is attributed to the PO_4_^3−^ group and it is characteristic to the structure of β-TCP.

The third spectrum, shown in Fig. 4c, was obtained for the AgHAp_40 powder after the thermal treatment at 1000 °C. It can easily be observed that this spectrum is very different from the other two spectra described earlier. Although there are some peaks associated to the apatitic structure, the one from 1024 cm^−1^ and the one from 602 cm^−1^, both of them describing the vibrations of phosphate group, it can be affirmed that the structure of this sample suffered major alterations caused by the thermal treatment temperature. In this context, it can be observed that some of the bands previously described have shifted, while others have disappeared completely and new ones have appeared. Therefore, the band initially found at 472 cm^−1^ has shifted to 431 cm^−1^, while the band initially found at 1089 cm^−1^ (Fig. 4a) which shifted to 1087 cm^−1^ (Fig. 4b) has shifted once again and is now found at 1080 cm^−1^ (Fig. 4c). The other bands, found at 549, 1119, 943, and 970 cm^−1^, are associated to the β-TCP structure. Thus, the bands from 943 and 1119 cm^−1^ are associated to the stretching vibrations of the PO_4_^3−^ group, while the band from 549 cm^−1^ characterizes the bending vibrations of the O-P-O bonds of the phosphate group [41, 42]. The band from 970 cm^−1^ is associated to the HPO_4_^2−^ group of the β-TCP structure [43].

The bands found in the spectral range 3550–3600 cm^−1^ attributed to the water lattice are presented in the top right corner of each spectrum. The vibrational bands from 3571, 3587, and around 3594 cm^−1^ are characteristic to the O-H vibrations [9, 44, 45]. Previous studies [9] have already proved that the band from 3571 cm^−1^ is characteristic to the hydroxyapatite phase. It can be observed that with the increase of the thermal treatment temperature, all the bands associated with the water lattice become narrower.

Comparing the three spectra presented in Fig. 4, it can be concluded that the thermal treatment has a major role in the structural alteration of the studied samples. A general widening of the vibrational bans can be observed as well as an increase of the intensity in the case of the band from 602 cm^−1^. The structure of the samples changes gradually with the increase of thermal treatment temperature, from poorly crystalline precipitated calcium phosphate (AgHAp_40) to predominant β-TCP structure (AgHAp_1000). In the AgHAp_600 sample, the presence of two phases, HAp and β-TCP, was highlighted.

Raman spectroscopy was used in order to obtain complementary information regarding the presence of the functional groups in the structure of poorly crystalline precipitated calcium phosphate AgHAp (*x*_Ag_ = 0.5) powders before (AgHAp_40) and after thermal treatment at 600 and 1000 °C. The Raman spectrum for the poorly crystalline precipitated calcium phosphate sample (AgHAp_40) was reported in our previous studies [2].

As we described in our previous research [2], in the Raman spectra of the samples obtained at room temperature, all the major vibrational bands characteristic to pure HAp structure are presented.

**Fig. 5:**
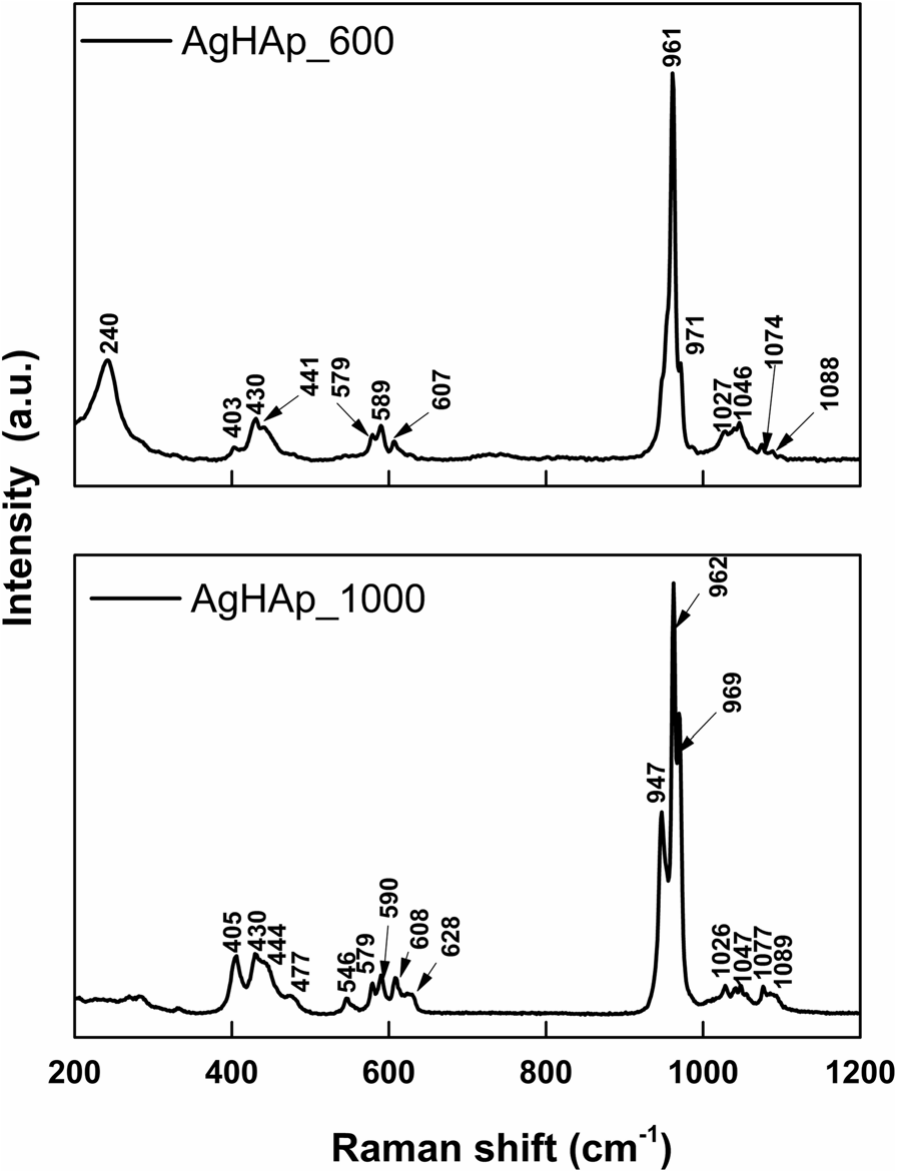
Raman spectra of AgHAp_600 and AgHAp_1000 samples

The Raman spectra obtained for the AgHAp_600 and AgHAp_1000 samples are presented in Fig. 5. In the case of AgHAp_600 powder, the Raman spectrum is dominated by the intense vibrational band at 961 cm^−1^ attributed to symmetric stretching mode (*ν*_1_) of the PO_4_^3−^ group from the HAp structure. Other vibrational bands associated to PO_4_^3−^ internal modes from the HAp structure are presented at 430 cm^−1^ (*ν*_2_), 441 cm^−1^ (*ν*_2_), 579 cm^−1^ (*ν*_4_), 590 cm^−1^ (*ν*_4_), 607 cm^−1^ (*ν*_4_), 1027 cm^−1^ (*ν*_4_), 1046 cm^−1^ (*ν*_4_), and 1074 cm^−1^ (*ν*_4_) [3, 29, 46].

The formation of a small quantity of β-tricalcium phosphate due to the thermal treatment at 600 °C is confirmed by the presence of the band from 971 cm^−1^ which is assigned to the stretching mode (*ν*_1_) of PO_4_ group from the β-TCP structure (Fig. 5a). Also, the band observed at 403 cm^−1^ could be associated to the O-P-O bending mode (*ν*_2_) of HPO_4_^2−^ group from the β-TCP structure [29]. Moreover, the presence of β-TCP in the samples is confirmed by the peak from 1088 cm^−1^ which is attributed to P-O stretching mode (*ν*_3_) of HPO_4_^2−^group [29].

In the case of AgHAp_1000 powder, the presence of β-TCP is highlighted by the presence of numerous characteristic vibrational bands. In the Raman spectrum of the sample thermal treated at 1000 °C (Fig. 5b), it is obvious that the intensity and the number of the bands characteristic to the HAp structure have decreased drastically. On the other hand, the intensity and the number of the bands characteristic to the β-TCP have increased significantly. The new bands observed at 477 cm^−1^ (*ν*_2_), 546 cm^−1^ (*ν*_4_), 628 cm^−1^ (*ν*_4_), and 947 cm^−1^ (*ν*_1_) are associated to the PO_4_^3−^ internal mode from the structure of β-TCP [46]. Moreover, the displacement and the smoothing of vibrational bands attributed to the phosphate group from the calcium-deficient hydroxyapatite structure were noticed.

According to [46], when the bands from the *ν*_2_ region are very close to the ones from the *ν*_4_ region, it means that the structure belongs to the β-TCP. Meanwhile, when the peaks from the ν_2_ region are clearly separated to the ones from the *ν*_4_ region, it means that the structure belongs to the HAp. This behavior was observed in our case, and it marks the major difference between the two samples (AgHAp_600 and AgHAp_1000).

The results obtained by Raman spectroscopy confirm the fact that the increase of the thermal treatment temperature of the powders led to the formation of a secondary phase. Also, it was observed that at 1000 °C, the powders became more crystalline.

The antimicrobial activity of the obtained powders was assessed against three microbial strains, representative for the Gram-negative, Gram-positive, and fungal species involved in the etiology of implant-associated diseases.

The antimicrobial activity evaluation results showed that the AgHAp composites (AgHAp_40, AgHAp_600, and AgHAp_1000) proved to be good antimicrobial activities against *S. aureus*, *K. pneumoniae*, and *C. albicans* microorganisms. In addition, it may be noted that the microbial activity was influenced by the thermal treatment of the samples.

In the qualitative assay, we have quantified the growth inhibition zone diameters induced after the deposition of 10 μL of the DMSO stock solution over the microbial culture. The used DMSO solvent did not influence the antimicrobial activity of the tested powders at the tested concentration.

**Fig. 6:**
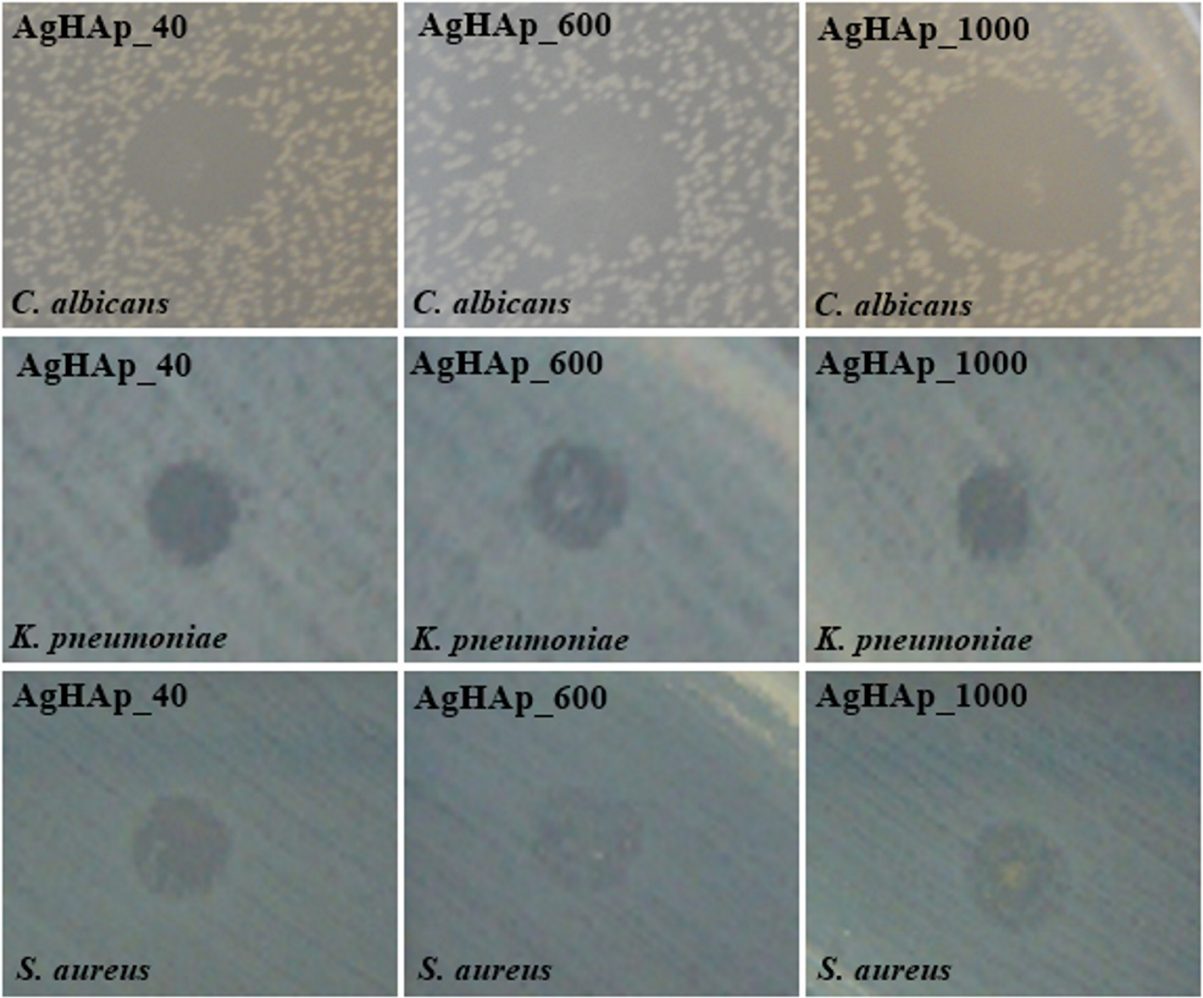
The antimicrobial activity of the tested compounds revealed by the presence of microbial growth inhibition zones

All tested powders proved to be active against the tested strains, the most susceptible one being *C. albicans*, on which all three tested powders exhibited a fungicidal effect, as revealed by the total inhibition of fungal growth on the area of the DMSO suspension diffusion (Fig. 6).

In the case of *K. pneumoniae* strain, only the silver-doped poorly crystalline precipitated calcium phosphate sample proved to have a bactericidal effect, while the ones thermally treated at 600 and 1000 °C were only bacteriostatic, as revealed by the presence of microbial colonies inside the inhibition zone (Fig. 6). In the case of *S. aureus*, all tested combinations exhibited a bacteriostatic effect (Fig. 6).

**Fig. 7:**
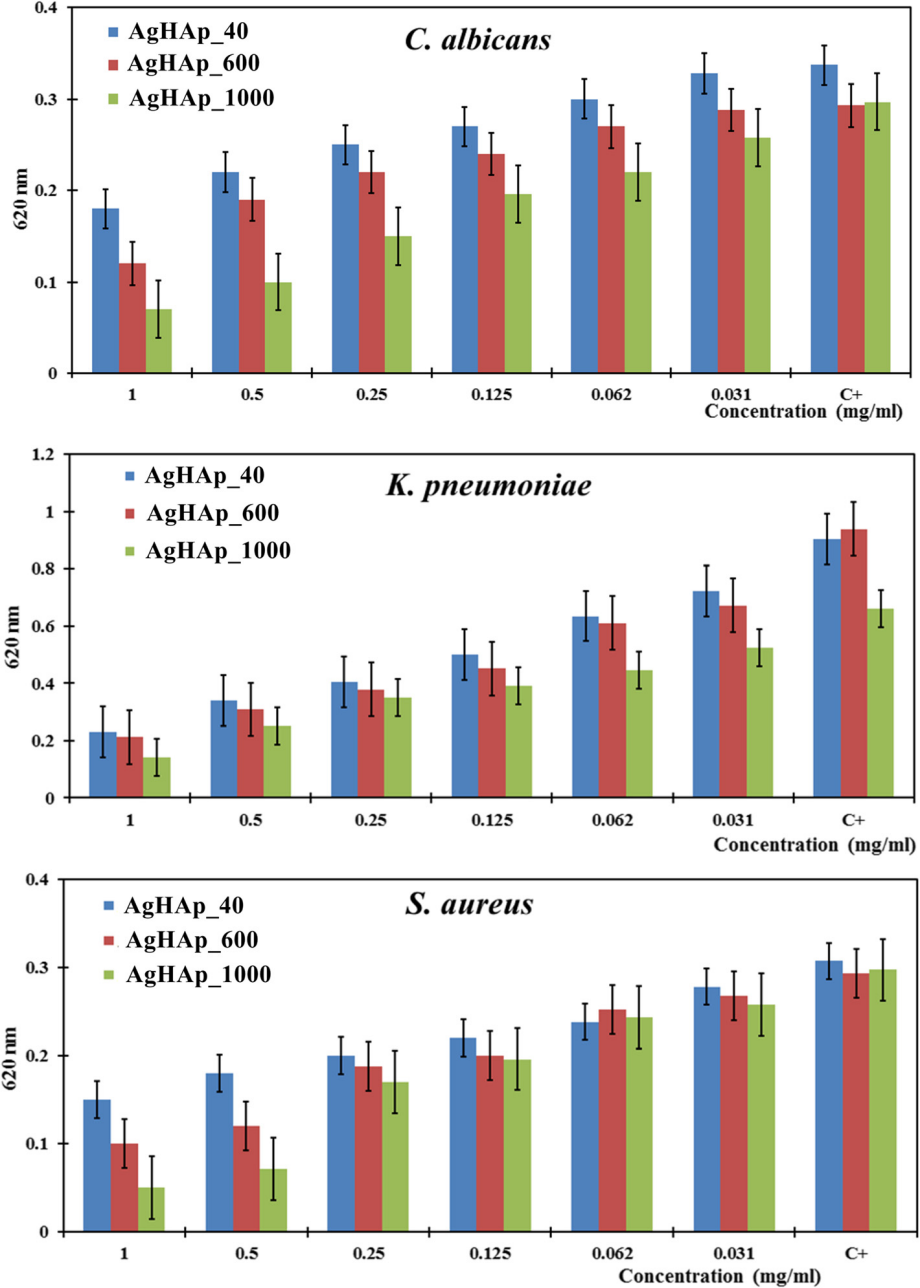
The microbial growth inhibition induced by different binary concentrations of the tested powders. The MIC (mg/mL) values were defined as the lowest concentration inhibiting the microbial growth as compared to the positive culture control

The quantitative assay of the antimicrobial activity of the tested powders revealed a dose-dependent intensity of the inhibitory effect, with the lowest values of the absorbance of microbial cultures (620 nm) recorded at the highest tested concentrations (Fig. 7). The intensity of the antimicrobial effect for all three tested powders against the microbial strains decreased in the following order of the thermal treatment temperature: 1000 > 600 > 40 °C.

However, the MIC values, defined as the lowest concentration inhibiting the microbial growth as compared to the positive culture control, were similar for the majority of the tested compounds, i.e., 0.031 mg/mL, excepting *C. albicans*, for which the AgHAp_40 and AgHAp_600 samples exhibited a higher MIC value, of 0.062 mg/mL (Table 2).

**Table 2:** The MIC (mg/mL) values of the tested powders against the tested microbial strains

Microbial strain	AgHAp_40	AgHAp_600	AgHAp_1000
*C. albicans*	0.062	0.062	0.031
*K. pneumoniae*	0.031	0.031	0.031
*S. aureus*	0.031	0.031	0.031

The silver-doped poorly crystalline precipitated calcium phosphate ceramic powders, AgHAp, may be used in the form of nanostructures for tissue engineering. This study aimed to investigate the changes induced by the thermal treatment on the structure, crystallinity, and shape of nanoparticles, as well as on their antimicrobial properties. Optical and structural investigations have shown that when the temperature increases, changes in the structure, morphology, and crystallinity of poorly crystalline precipitated calcium phosphate AgHAp nanopowders occur. On the other hand, an increase of the β-TCP could be observed when the temperature at which the samples were subjected to heat treatment increased. The presence of β-TCP in the samples after thermal treatment before 1300 °C might be explained by a minor imbalance that occurs in the stoichiometric ratio (the standard value for molar ratio of Ca/P is 1.67). According to previous studies presented by Berzina-Cimdina and Borodajenko [9], inclusion of impurities, often substitutions of Ca^2+^ or interpenetration of other ions in the crystal lattice, could be one of the main reasons of non-stoichiometry. Furthermore, depending on the Ca/P molar ratio, it is possible to obtain numerous calcium phosphates of different compositions (HAp, β-TCP, or HAp and β-TCP mixture). The Ca/P molar ratio is connected to the pH of the solution. The Ca/P molar ratio determined for AgHAp_40 powder was 1.66. This value is characteristic to HAp but a partial conversion from HAp to β-TCP was observed after thermal treatment at 600 and 1000 °C of AgHAp_40 powder. More than that, the thermal treatment caused an increase of the β-TCP content in the powders depending on the thermal treatment temperature. A decrease of the Ca/P atomic ratio to 1.63 and 1.58 for the AgHAp_600 and AgHAp_1000 powders was also observed. The Ca/P atomic ratios for the AgHAp_600 and AgHAp_1000 powders do not coincide with the stoichiometric ratios of HAp (1.67) and β-TCP (1.50), respectively. The same behavior was observed by Boutinguiza et al. [47] for calcium phosphate-based materials of marine origin. These results are in good agreement with previous studies conducted by Piccirillo et al. [48]. In their studies, on silver-containing calcium phosphate materials, Piccirillo et al. [48] noted that Ag-containing samples have lower Ca/P ratios. Nevertheless, the values of Ca/P molar ratios are greater than expected for the biphasic materials, showing the presence of a biphasic material with non-stoichiometric phases. According to Piccirillo et al. [48], we can conclude that beyond mere ion exchange, several different processes can take place during the thermal treatment leading to various modifications in the structure, composition, and, therefore, the final material. In accord with Dorozhkin [10–12], the HAp (Ca/P = 1.67), β-TCP (Ca/P = 1.5), and biphasic calcium phosphate, which mainly consists of a mixture of HAp and β-TCP in various ratios, are most frequently used for biomedical application.

As it was observed in this study, the conversion from HAp to β-TCP does not compromise the validity of the material for biomedical applications in agreement with previous studies [48]. Moreover, increasing the amount of TCP in the powders does not undermine the antimicrobial activity of the powders (AgHAp_600 and AgHAp_1000). More than that, all the samples show better efficacy towards Gram negative bacterial strains such as *K. pneumoniae 11* than Gram-positive *S. aureus ATCC 6538* bacterial strains or *C. albicans ATCC 10231* fungal strain. Our results confirmed the studies conducted by Piccirillo et al. [48] but the study of antimicrobial activity induced by silver in HAp structure remains an open field. The complexity of the mechanisms of silver antibacterial action is demonstrated by various studies against Gram-negative, Gram-positive, and fungal strains. Stanić et al. [49] in their studies on the synthesis of antimicrobial monophase silver-doped hydroxyapatite nanopowder showed that the Gram-negative strains were more sensitive. At the same time, Dorozhkin [50] and Radovanović et al. [15] in their studies on the antimicrobial activity and biocompatibility of Ag^+^-doped biphasic, triphasic, and multiphasic calcium orthophosphates established that these materials were more effective against Gram-positive strains. Therefore, it was observed that a temperature increase led to a significant improvement of the antimicrobial properties, the materials resulted after thermal treatment at 600 °C and 1000 °C being thus more adequate for being used in orthopaedic and dental applications, due to their superior ability to prevent infections that may occur in vivo. Whereas, the mechanisms of antibacterial action of the silver are quite complex; it requires detailed studies to establish the optimal doses of silver that can be used in various treatments (orthopaedic infections or various infected wounds) without causing side effects.

## Conclusions

The results presented in this paper highlight the influence of thermal treatment on the physicochemical and antimicrobial properties of the silver-doped poorly crystalline precipitated calcium phosphate (AgHAp) powders, when the silver concentration is *x*_Ag_ = 0.5. The XRD studies revealed that the structure and the crystallinity of the samples change gradually with the increase of thermal treatment temperature. Therefore, the structure of the samples changed from poorly crystalline precipitated calcium phosphate to predominant β-TCP, becoming at the same time more crystalline when the temperature increased from 600 to 1000 °C. In terms of morphology, the shape and dimension of the nanoparticles began to change when the thermal treatment temperature increased. The FTIR and Raman spectroscopy studies revealed that the structure of the samples also changed gradually with the thermal treatment temperature, from silver-doped poorly crystalline precipitated calcium phosphate (AgHAp_40) to a β-TCP predominant structure (AgHAp_1000). In the AgHAp_600 sample, the presence of two phases, poorly crystalline precipitated calcium phosphate and β-TCP, was revealed. Regarding the antimicrobial activity, the materials resulted after thermal treatment at 600 °C and 1000 °C presented improved properties, being thus able to resist to microbial colonization and preventing subsequent infections caused by Gram-positive (*S. aureus*), Gram-negative (*K. pneumoniae*), and fungal (*C. albicans*) microorganisms.
